# Recommendations for Ethical Review of Veterinary Clinical Trials

**DOI:** 10.3389/fvets.2021.715926

**Published:** 2021-07-28

**Authors:** Jessica A. Bertout, Philippe J. R. Baneux, Carol K. Robertson-Plouch

**Affiliations:** ^1^Companion Animal Studies for Translational Research Alliance, Inc., Issaquah, WA, United States; ^2^Center for Animal Resources and Education, Cornell University, Ithaca, NY, United States; ^3^Convergence Bioscience, Indianapolis, IN, United States

**Keywords:** ethical review, clinical trials, translational research, comparative medicine, medical research, drug development, one health, veterinary clinical studies committee

## Abstract

Ethical review of both human and animal research is critical to ensuring that studies are conducted with due regard to the welfare and safety of enrolled subjects and to the integrity of the data. However, differences exist in laws, policies, and best practices between human and animal studies. Ethical review is required for most human studies. While the laws and standards are clear for humans and for laboratory animals, the laws and standards for clinical research for client-owned animals are not as well-defined. Here, we discuss gaps in ethical review of clinical animal research in the United States of America and propose expanded functions for veterinary clinical studies committees as a solution.

## Introduction

Biomedical research involves progressive and iterative information gathering. Following initial laboratory studies, clinical research studies in human participants are critical to confirming safety and efficacy of drugs and devices and to successfully translating basic science into novel treatments, techniques, and solutions used in standard medical practice. The same can be said for veterinary medicine, with clinical studies in privately-owned pets representing a crucial step toward establishing safety and effectiveness in the development of drugs for companion animals. In addition, because companion animal species naturally develop a number of diseases or conditions that parallel those found in humans, studies involving privately-owned pets can help fuel advances in both human and veterinary medicine. Some examples of this cross-species research approach include comparative studies in oncology (1), orthopedics (2), pain (3), immunology (4), aging (5), as well as neurologic and genetic diseases (6–8). Several recent articles review the many benefits of including translational studies with veterinary patients in biomedical research and product development plans (9–11). Herein, “veterinary clinical studies” refer to clinical studies enrolling privately-owned companion animal patients (pet dogs, cats, horses, etc.,) through veterinary clinics or hospitals, similar to clinical trials with human participants. The term does not refer to studies in laboratory animals owned by a research facility.

Whether focused on human or animal patients, documented ethical review of clinical studies by a structured review committee is of paramount importance. It provides confirmation to the individuals proposing the research that their study meets high scientific, ethical, and quality standards that justify the investment of resources. In addition to providing an expert and multifaceted opinion on the ethical and scientific soundness of the study, this review also infers confidence to multiple other parties: to the clinicians referring patients to the studies, to the investigators conducting the studies, and to the participants/subjects (and their parents, guardians, or owners) in the study. Finally, the incorporation of an ethical review is key to reassuring the public that the research studies have been designed and will be conducted with the utmost regard to the safety and well-being of humans and/or animals. Given that clinical studies are of key importance to advancing knowledge in biomedical research, regardless of the species enrolled in the research, oversight that can provide review of study design, informed consent, and potential risks is essential.

This paper addresses gaps and potential solutions for ethical review of clinical studies in animals, while recognizing the need for a streamlined approach that will minimally burden researchers, regulators, and sponsors, but still ensure protection of the animals and informed consent of the owners.

## Review

### Differences in Laws and Regulations for Human and Animal Clinical Research

Ethical conduct of research with human and animal subjects is a continuously evolving topic. Across time, research tragedies have influenced and informed professional policy, guidelines, and laws worldwide, leading to pivotal documents on the ethical conduct of human research, including the *Nuremburg Code* (12), the *Declaration of Helsinki* (13), and *The Belmont Report* (14). First published in 1978, *the Belmont Report* outlines principles of ethical human research in the United States (U.S.), demands informed consent for all study participants, and requires ethical review board oversight for human clinical studies, with certain exemptions for research involving no more than minimal risk. The law also defines the role, responsibilities, and composition of the ethical review board (15).

For veterinary clinical studies with client-owned animals, the laws pertaining to research are scant. While individual state laws govern the practice of veterinary medicine, the conduct of clinical research involving privately-owned pets is typically not addressed in these state laws. The Animal Welfare Act (AWA) (16), first enacted in 1966, “sets general standards for humane care and treatment that must be provided for certain animals that are bred for commercial sale, sold sight unseen (Internet sales), exhibited to the public, used in biomedical research, or transported commercially” [USDA The Blue Book, Introduction Page 1 (17)].

Enforcement of the AWA Law is assigned to the U.S. Department of Agriculture (USDA) (17). The USDA administers the AWA Regulations, which govern how the AWA Law will be enforced by the USDA. However, the AWA Law and the AWA Regulations do not address the ethical management of clinical studies that enroll client-owned animals such as pet dogs, cats, or horses.

In addition to the AWA, the Health Research Extension Act of 1985, Public Law 99-158, “Animals in Research” (November 20, 1985), provides the statutory mandate that informs use of animals in federally funded research. In the same year, the Interagency Research Animal Committee promulgated the “U.S. Government Principles for the Utilization and Care of Vertebrate Animals Used in Testing, Research and Training (Principles).” In 1986, these Principles were incorporated by the U.S. Public Health Service (PHS) into the Policy on Humane Care and Use of Laboratory Animals. PHS policy is administered by the Office of Laboratory Animal Welfare (OLAW) and requires an Assurance from the institution in order to receive PHS/National Institutes of Health (NIH) funds to conduct research with animals. The PHS policy requires the use of the *Guide for the Care and Use of Laboratory Animals* (18) as the reference standard for the research program. This document was first written to provide research institutions with consistent guidance for the care, use, and ethical treatment of animals involved in research. As with the AWA, the focus of these regulations and guidance is limited to animals used in laboratory research or in commercial settings (as described in the AWA Law and AWA Regulations) and lacks specific reference to privately-owned companion animals whose owners may wish to enroll them in clinical studies.

### Regulatory Agencies

The Food and Drug Administration (FDA) is the primary agency overseeing human drug development in the U.S., and includes subsections such as the FDA Center for Drug Evaluation and Research (CDER), the FDA Center for Biologics Evaluation and Research (CBER), and the FDA Center for Devices and Radiological Health (CDRH), which oversee the respective categories of drug, biologic, device-based and radiologic interventions (19, 20). For animal health drug development, the FDA Center for Veterinary Medicine (CVM) is a central organization, with subgroups within the CVM focused on specific categories. All of these FDA centers are given this authority through the Federal Food, Drug, and Cosmetic Act [FFDCA (21)]. For animal health, besides the FDA CVM, there are two other key agencies that can be the authority in the approval and regulation of animal health product development; the USDA Center for Veterinary Biologics (CVB) and the Environmental Protection Agency (EPA) (22).

The USDA CVB administers the process for approval of many biologics for animal health. The USDA CVB regulates these as per the law outlined in the Virus-Serum-Toxin Act (VSTA), established in 1913 and revised in 1985 (23, 24). The USDA CVB has evolved guidance with issuance of a series of Veterinary Services (VS) Memoranda. For example, VS Memorandum 800.126 “Efficacy and Safety Studies for Cancer Immunotherapeutics” was issued in September 2020 with the advancement of more biologic products as oncologic and immunologic therapeutic agents (25).

Since animal biological products are “drugs” within the meaning of the FFDCA, dialogue can become necessary between the USDA CVB and the FDA CVM. This dialogue becomes even more relevant with the advent of new technologies and development of some biologics as therapeutic agents (rather than only preventive agents). The FDA CVM and USDA CVB have established the Memorandum of Understanding 225-05-7000, which outlines the process by which the two agencies determine the appropriate jurisdiction for approval and regulation of potential new products that may come into question, stating “…the FDA regulates such products when they are not produced and distributed in full conformance with the VSTA and its implementing regulations” (26).

The EPA can also be involved as an approval agency for animal health products when substances are being evaluated for external application for control of pests, such as fleas, ticks and other infestations. The standards by which the EPA regulates these substances are found in the Federal Insecticide, Fungicide, and Rodenticide Act (FIFRA). The EPA also regulates tolerance levels for pesticide residues on food products (27).

### Good Clinical Practice Standards for Clinical Studies

In 1990, the first meeting of the International Council on Harmonization of Technical Requirements for Pharmaceuticals for Human Use (28) occurred in Brussels. In 1996, the Veterinary International Conference on Harmonization (VICH) was established. The VICH is a trilateral organization led by the U.S., the European Union, and Japan (29), which continues to expand toward global support and participation. One of the objectives of the VICH is to “establish and implement harmonized technical requirements for the registration of veterinary medicinal products in VICH regions” (30). Both the ICH and VICH have been important sources of internationally accepted standards. As an example, the ICH Good Clinical Practice (GCP) and the VICH GCP (VICH GL9) were developed to provide harmonized guidance and standards of conduct for sponsors and investigators involved in clinical studies. These standards are often referenced in regulatory guidance documents.

The VICH GCP specifically covers clinical research studies involving both food-producing animals and companion animals and is integrated into U.S. Food and Drug Administration (FDA) Center for Veterinary Medicine (CVM)'s guidance for conduct of animal health research (31). Clinical studies focused on investigational drugs that have an open INAD with the FDA or an open VBPL with the USDA are subject to regulatory requirements enforced by these organizations. However, VICH GCP are not consistently applied or required for clinical studies that are conducted outside the scope of an FDA CVM submission or other regulatory obligation. For example, USDA Memorandum 801-301 (2001) (25) presents the VICH GCP concepts but also indicates that an alternative approach can be used if it satisfies the applicable statute and regulations (32).

### Gaps in Ethical Review of Veterinary Clinical Studies

When veterinary clinical studies are conducted at privately owned veterinary clinics, the requirement for, and availability of, scientific or ethical review is often lacking. While many academic and industry institutions require Institutional Animal Care and Use Committee (IACUC) review of all animal studies regardless of funding source or regulatory oversight, veterinarians in private practice often do not have access to an IACUC or any other institutional or internal ethical review board. As such, private veterinary hospitals sometimes face hurdles when initiating or conducting clinical studies and must rely on the ethical review conducted by other participating centers or the sponsor, convene their own review panel, or end up having to forego an ethical review altogether.

Reduced access to ethical review can present a serious challenge for veterinary medical doctors in private practice wishing to conduct and publish research. Many journals justifiably require a statement from the authors confirming that the research being submitted for publication was approved by an ethical review committee prior to study conduct. Consequently, in the absence of an ethical review, veterinary medical doctors employed in private practice may face rejection of their original research manuscripts by journals. To further complicate this substantial conundrum, those doctors who are completing residencies in private practices are often applying for specialty board certification; these boards typically have a first-author publication requirement as part of the credentialing process (33). Without access to ethical review, these residents may therefore, as an unintended consequence, face delays to attaining their board certifications.

As mentioned above, some institutions rely on the IACUC to oversee all research involving animals, including clinical research involving privately-owned pets; but there are limitations to this approach. These limitations stem from the fact that IACUCs were established to assure oversight of animal care and use programs at research institutions, with focus on laboratory animals, appropriate housing and care, facility inspections, and administrative structures and processes. However, the AWA laws and regulations and the IACUC review do not address some of the most critical elements of clinical research, including, as an example, the process of informed consent. Yet, as in human clinical research, informed consent is a tenet of ethical research in animal patients (11, 34, 35), should be common practice in veterinary medicine, and is required procedure in veterinary practice under some state laws (36). There are both similarities and differences in the informed consent in human studies and in animal studies, with animal studies bearing a distinction that consent is by the owner, rather than the human participant or parent of a pediatric patient. Gaps exist within the informed consent process for human, pediatric, and veterinary patients, but those are beyond the scope of this publication.

### Veterinary Clinical Studies Committee

Recognizing some of the gaps discussed above, the AVMA proposed a policy in 2013, describing the need for oversight of studies involving client-owned animals, and suggesting that a Veterinary Clinical Studies Committee (VCSC) be formed (37). The AVMA, with membership at nearly 97,000 in 2020, is the largest representative membership of veterinarians in the U.S.; however, individual states regulate the license to practice veterinary medicine, and membership in the AVMA is not required. The AVMA policy indicates that the VCSC should ensure that informed consent is obtained from owners and that the animals enrolled in the studies are protected from conflict-of-interest issues. Further, the AVMA guidance indicates that the VCSC should be composed of veterinarians primarily involved in clinical practice and should work cooperatively with the IACUC. The Policy does not currently address situations in which an IACUC is not currently available, as in many private practices. While the AVMA policy is an important reference document, it does not constitute an enforceable legal requirement and provides limited guidance to the research community on standards to be applied for such a review process. The United Kingdom has published a similar, also non-binding document which compiles the recommendations of a working group (38). The Royal College of Veterinary Surgeons (RCVS) began providing an “Ethics Review Panel” for its practice-based members that do not have other access to ethical review of veterinary clinical trials (39), following a January 2019 meeting of the RCVS Council.

Human clinical trial processes offer additional insight. As discussed above, ethical review of human clinical research conducted in the U.S. has been required for many years. Moreover, with the growth in numbers of large, complex, multi-center human trials in the United States over the past few decades, the FDA has recognized the increased burden placed on individual institutional review boards (IRBs) and sponsors, which can result in delays to the conduct of research. Consequently, there has been a shift away from individual reviews by each site's IRB toward the use of a single centralized IRB for each multi-center study. Instead of working with IRBs at each of the clinical sites participating in a trial, each with different institutional processes and turnaround times, the study documents are submitted to a single centralized IRB, approved by those clinical sites to conduct the reviews, thus, reducing duplication of effort and improving efficiency. The FDA guidance on the use of a centralized IRB provides that “A centralized IRB review process involves an agreement under which multiple study sites in a multicenter trial rely in whole or in part on the review of an IRB ***other***than the IRB affiliated with the research site,” thus, allowing local and central IRBs to enter into arrangements appropriate for the clinical research under review (40). Further, the U.S. Health and Human Services, in the opening Background statement on their policy regarding Single IRB Exception Determination, states “The revised Common Rule (i.e., the 2018 Requirements) requires at 45 CFR 46.114(b) that all institutions located in the United States that are engaged in cooperative research conducted or supported by a Federal department or agency rely upon approval by a single IRB for the portion of the research that is conducted in the United States,” and provides some guidance on the determination for exception to single IRB requirements (41). The centralized IRB may be either an institutional board or an independent entity.

The central review concept for multi-site studies can be useful for veterinary studies as well. Consistent with this notion, a consortium of veterinary schools has suggested that a centralized IACUC (with incorporation of VCSC concepts) could promote more efficient start-up timelines for studies conducted within participating academic institutions (42). However, for private, non-academic veterinary practices, there are limited options available. The creation of one or multiple independent VCSCs could provide this critical support. These VCSCs could be designed as for-profit and/or non-profit entities, supported by key stakeholders, and accessible to all sponsors and investigators, including those in private practice or pharmaceutical and biotechnology industries. We propose that the VCSC be responsible for completing the review and approval of (1) the final study documents (including protocol and informed consent) prior to initiation of the study; (2) any amendments to study protocols or other significant changes during the study; (3) reports of any serious and unexpected adverse events throughout the course of the study, and ensuing actions, if applicable; (4) updated safety information as it becomes available; (5) any as-needed updates for other reasons, including critical staff changes or other concerns; and (6) a regular review of the protocol progress, at a frequency determined by the VCSC (including case enrollment status, adverse events, and protocol deviations). This process may be iterative, as the committee will request additional information, changes, or resubmission in order for the study to meet the approval criteria.

**Figure 1:**
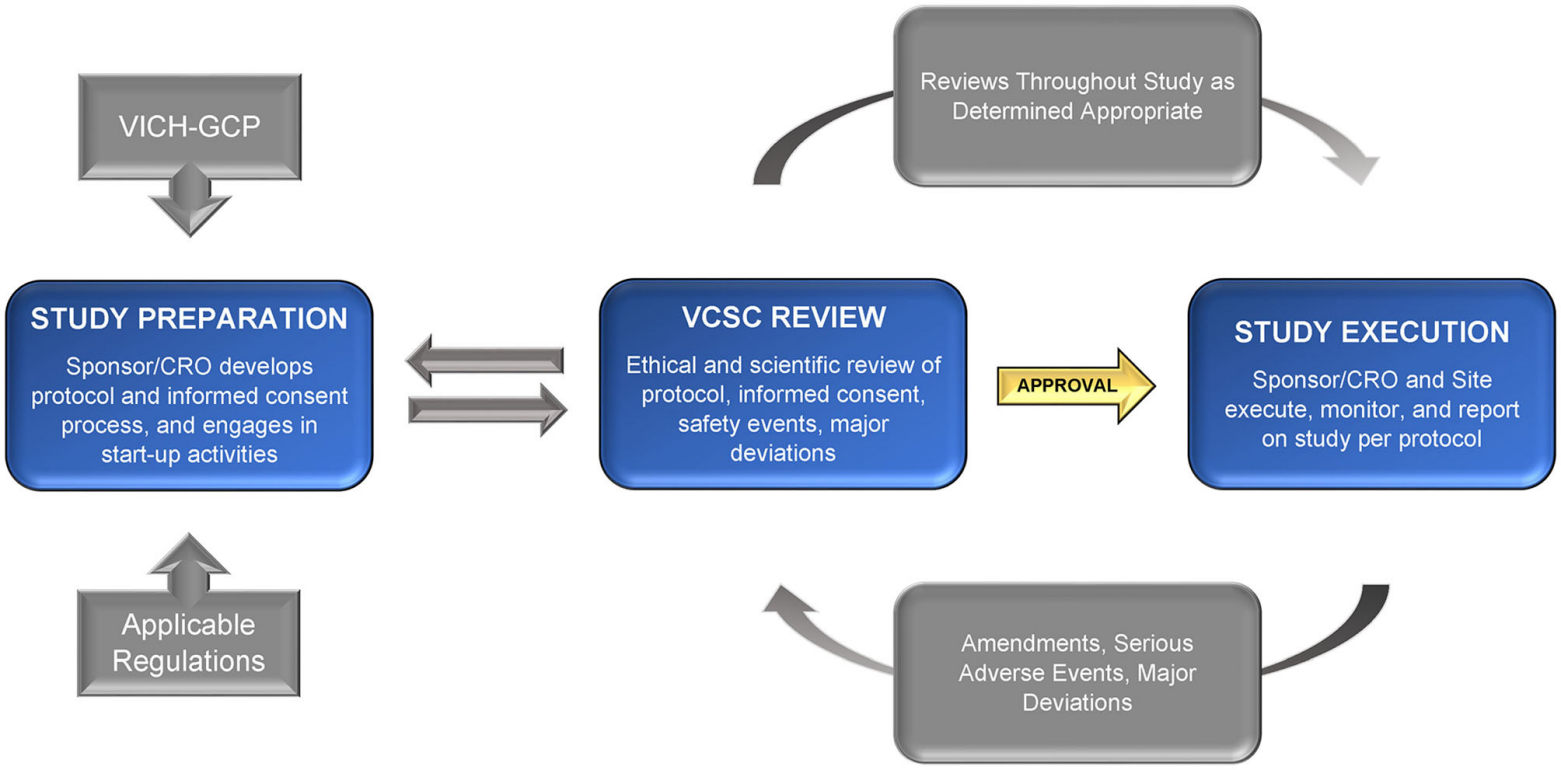
Flowchart: ethical review process for veterinary clinical studies.

Among tasks outside the scope of the VCSC would be those typically managed by the sponsor or Contract Research Organization (as described in the VICH GCP), including qualifying veterinary sites, ensuring appropriate training of study staff, monitoring data, or verifying continued protocol compliance. The VCSC should instead be focused on the ethical and scientific merits of the protocol, the informed consent document and process, the continued welfare and safety of the research subjects enrolled on the protocol, and the safety of treatment administrators or others involved. Refer to Figure 1 for a graphic overview of the process.

We propose that the optimal VCSC review board composition and charter reflect the goals of the ethical review, including ensuring safety and welfare of the animals to be enrolled. To achieve this, we suggest the VCSC include members representing traditional IACUC roles, as well as specialists, who can address clinical research concerns outside the scope of laboratory animal studies. In this way, the VCSC would provide appropriate ethical review of clinical studies and would do so even for studies conducted for entities that do not have an IACUC. The proposed review committee would include a member with training and experience in laboratory animal science and medicine, as those specialists are trained extensively in the regulations, ethics, quality assurance standards and record keeping surrounding the use of animals in research. The committee would also include at least one practicing scientist, at least one non-scientist member representing the general community at large, and at least one veterinarian (preferably more) primarily involved in clinical practice; including, ideally, at least one member with experience in the area of the clinical study under review.

In addition, the committee may include *ad hoc* reviewers based on each study's focus area and needs. To achieve this, the organizers of the committee would have a network of expertise such as biostatisticians and veterinary specialists who are qualified, trained, and available to review studies upon request. For example, this network could include a radiation oncologist for review of a study focused on the effects of a specific new radiation protocol on canine osteosarcoma, or a veterinary dermatologist to review a study concerning a new antipruritic agent. For translational studies that could benefit both human and animal patients, a representative from the corresponding human medical field of interest could also add value.

All committee members should be trained on VICH GCP, The Guide for the Care and Use of Laboratory Animals, the Guide for the Care and Use of Agricultural Animals in Research and Teaching (when appropriate), applicable items in the U.S. Government Principles for the Utilization and Care of Vertebrate Animals used in Teaching, Research and Training (43), any relevant state and federal regulations, and awareness of current concerns in bioethics relevant to clinical research in animals. Based on their role and expertise, certain committee members should remain current on commonly accepted professional standards (standard of care) as reflected in the contemporary scientific literature.

To be most impactful, the review process would need to be efficient, clearly defined, and accessible with an appropriate fee structure. Systems should be established to clarify the roles of the Investigators, Sponsors, and the Review Board, communicate expectations, and provide relevant training to all concerned parties. Standardization of processes will increase the efficiency, thoroughness, and objectivity of the ethical review, and drive toward positive outcomes. Finally, review committee accreditation by an independent entity or organization would be highly beneficial to ensure that minimum review process standards are met and maintained, and that confidence within the scientific field, the veterinary community, and the public is ensured.

## Conclusion

Veterinary clinical studies are important not only for animals but also for their contributions to translational science; however, ethical review requirements need better definition. The value of the comparative medicine link is increasingly recognized for its impact on new developments in human and animal health. Veterinary clinicians in private practice and private sponsors of veterinary studies should be encouraged in these endeavors, similarly to those in the academic environment. Providing an accessible process to perform ethical review for clinical studies regardless of the professional setting will support this important work. Similarly, including clinical research in veterinary training would strengthen the role and impact of veterinary medicine in the One World One Health concept.

## Author Contributions

All authors listed have made a substantial, direct and intellectual contribution to the work, and approved it for publication.

## Conflict of Interest

JB co-owns a veterinary clinical research organization (CASTR Alliance, Inc.) that provides clinical research services, including ethical review, for pharma- or biotech- sponsored veterinary clinical studies. JB also has an equity stake in and is a consultant for Presage Biosciences, Inc. PB is the chair of CASTR Alliance, Inc.'s Veterinary Clinical Studies Committee, is a member of the AVMA Council on Research and a member of the VCSC at Cornell University, Ithaca, NY. CR-P provides biotech and bio/pharmaceutical development services for human and animal applications through Convergence Bioscience, LLC, is a member of the Indiana University Institutional Review Board and is on the Board of the comparative oncology Canines-N-Kids Foundation and receives benefits from Lilly (LLY) and is a stockholder of numerous healthcare-related companies.

## Publisher's Note

All claims expressed in this article are solely those of the authors and do not necessarily represent those of their affiliated organizations, or those of the publisher, the editors and the reviewers. Any product that may be evaluated in this article, or claim that may be made by its manufacturer, is not guaranteed or endorsed by the publisher.

## References

[1] LeBlanc AK Mazcko CN. Improving human cancer therapy through the evaluation of pet dogs. Nat Rev Cancer. (2020) 20:727–42. 10.1038/s41568-020-0297-3 32934365

[2] Meeson RL Todhunter RJ Blunn G Nuki G Pitsillides AA. Spontaneous dog osteoarthritis - a One Medicine vision. Nat Rev Rheumatol. (2019) 15:273–87. 10.1038/s41584-019-0202-1 30953036 PMC7097182

[3] Robertson-Plouch C Stille JR Liu P Smith C Brown D Warner M. A randomized clinical efficacy study targeting mPGES1 or EP4 in dogs with spontaneous osteoarthritis. Sci Transl Med. (2019) 11:eaaw9993. 10.1126/scitranslmed.aaw9993 31666405

[4] Bilgic Temel A Murrell DF. Pharmacological advances in pemphigus. Curr Opin Pharmacol. (2019) 46:44–9. 10.1016/j.coph.2019.01.001 30974409

[5] Hoffman JM Creevy KE Franks A O'Neill DG Promislow DEL. The companion dog as a model for human aging and mortality. Aging Cell. (2018) 17:e12737. 10.1111/acel.12737 29457329 PMC5946068

[6] Golubczyk D Malysz-Cymborska I Kalkowski L Janowski M Coates JR Wojtkiewicz J. The role of glia in canine degenerative myelopathy: relevance to human amyotrophic lateral sclerosis. Mol Neurobiol. (2019) 56:5740–8. 10.1007/s12035-019-1488-3 30674036 PMC6614142

[7] Potschka H Fischer A von Ruden EL Hulsmeyer V Baumgartner W. Canine epilepsy as a translational model? Epilepsia. (2013) 54:571–9. 10.1111/epi.12138 23506100

[8] Steinmetz S Tipold A Loscher W. Epilepsy after head injury in dogs: a natural model of posttraumatic epilepsy. Epilepsia. (2013) 54:580–8. 10.1111/epi.12071 23294259

[9] Regan D Garcia K Thamm D. Clinical, pathological, and ethical considerations for the conduct of clinical trials in dogs with naturally occurring cancer: a comparative approach to accelerate translational drug development. ILAR J. (2018) 59:99–110. 10.1093/ilar/ily019 30668709 PMC6927823

[10] Garden OA Volk SW Mason NJ Perry JA. Companion animals in comparative oncology: One Medicine in action. Vet J. (2018) 240:6–13. 10.1016/j.tvjl.2018.08.008 30268334

[11] Page R Baneux P Vail D Duda L Olson P Anestidou L. Conduct, oversight, and ethical considerations of clinical trials in companion animals with cancer: report of a workshop on best practice recommendations. J Vet Intern Med. (2016) 30:527–35. 10.1111/jvim.13916 26950524 PMC4913608

[12] The Nuremberg Code 1947. BMJ. (1996) 313:1448. 10.1136/bmj.313.7070.1448

[13] World Medical Association. World Medical Association declaration of Helsinki: ethical principles for medical research involving human subjects. JAMA. (2013) 310:2191–4. 10.1001/jama.2013.281053 24141714

[14] U.S. The National Commission for the Protection of Human Subjects of Biomedical Behavioral Research. The Belmont Report: Ethical Principles Guidelines for the Protection of Human Subjects of Research. Bethesda, MD: The Commission; Washington: for sale by the Supt. of Docs., U. S. Govt. Print. Off. (1978).

[15] Adashi EY Walters LB Menikoff JA. The belmont report at 40: reckoning with time. Am J Public Health. (2018) 108:1345–8. 10.2105/AJPH.2018.304580 30138058 PMC6137767

[16] AWA. Animal Welfare Act. (2012). Available online at: https://uscode.house.gov/view.xhtml?path=/prelim@title7/chapter54&edition=prelim (accessed July 10, 2021).

[17] United States Department of Agriculture Animal and Plant Health Inspection Service. Blue Book. Animal Care: Animal Welfare Act and Animal Welfare Regulations. Washington, DC (2017). Available online at: https://awahistory.nal.usda.gov/search/5969370 (accessed July 9, 2021).

[18] National Research Council. Guide for the Care and Use of Laboratory Animals: Eighth Edition. (2011). Available online at: https://www.nap.edu/catalog/12910/guide-for-the-care-and-use-of-laboratory-animals-eighth (accessed July 10, 2021).

[19] FDA. FDA Organization. Available online at: https://www.fda.gov/about-fda/fda-organization (accessed June 21, 2021).

[20] FDA. From an Idea to the Marketplace: The Journey of an Animal Drug through the Approval Process. Available online at: https://www.fda.gov/animal-veterinary/animal-health-literacy/idea-marketplace-journey-animal-drug-through-approval-process (accessed June 21, 2021).

[21] US Congress. U.S. Federal Food, Drug, and Cosmetic Act (FD&C Act). Available online at: https://www.fda.gov/regulatory-information/laws-enforced-fda/federal-food-drug-and-cosmetic-act-fdc-act (accessed July 10, 2021).

[22] Animal Health Institute. Regulation of Animal Medicines. Available online at: https://ahi.org/approval-and-regulation-of-animal-medicines/ (accessed June 21, 2021).

[23] USDA. Virus-Serum-Toxin Act. 21 USC 151-159 et. seq. Available online at: https://www.aphis.usda.gov/animal_health/vet_biologics/publications/vsta.pdf (accessed July 10, 2021).

[24] Monke J. The Virus-Serum-Toxin Act: A Brief History and Analysis. (2005). Available online at: https://nationalaglawcenter.org/wp-content/uploads/assets/crs/RS22014.pdf (accessed July 10, 2021).

[25] USDA. VS Memorandum 800.126 Efficacy and Safety Studies for Cancer Immunotherapeutics. (2020). Available online at: https://www.aphis.usda.gov/animal_health/vet_biologics/publications/memo800-126.pdf (accessed July 10, 2021).

[26] USDA-APHIS and FDA. MOU 225-05-7000. (2021). Available online at: https://www.fda.gov/about-fda/domestic-mous/mou-225-05-7000 (accessed July 10, 2021).

[27] EPA. Summary of the Federal Food, Drug, and Cosmetic Act. (2002). Available online at: https://www.epa.gov/laws-regulations/summary-federal-food-drug-and-cosmetic-act (accessed July 10, 2021).

[28] ICH. About the International Council for Harmonization. Available online at: https://www.ich.org/page/history (accessed May 17, 2021).

[29] Alvarez JC Fortsch GW. Harmonization of regulatory requirements for the development of veterinary clinical trials–a drive toward globalization and simplification. Food Drug Law J. (2005) 60:407–11. Available online at: https://www.jstor.org/stable/26660286?seq=1#metadata_info_tab_contents (accessed July 10, 2021). 16304746

[30] VICH. What is VICH? Available online at: https://vichsec.org/en/about/what-is-vich.html (accessed May 17, 2021).

[31] FDA-CVM. Guidance for Industry #85: Good Clinical Practice - VICH GL9. Laurel, MD: Food and Drug Administration, Center for Veterinary Medicine (2001).

[32] USDA. Veterinary Services Memorandum NO. 800.301. (2001). Available online at: https://www.aphis.usda.gov/animal_health/vet_biologics/publications/memo_800_301.pdf (accessed July 10, 2021).

[33] Birkenheuer AJ Royal KD Cerreta A Hemstreet D Lunn KF Gookin JL. Perceptions and attitudes of Small Animal Internal Medicine specialists toward the publication requirement for board certification. J Vet Intern Med. (2020) 34:574–80. 10.1111/jvim.15717 32030794 PMC7096663

[34] Mandal J Parija SC. Informed consent and research. Trop Parasitol. (2014) 4:78–9. 10.4103/2229-5070.138533 25250226 PMC4166807

[35] Grimm H Bergadano A Musk GC Otto K Taylor PM Duncan JC. Drawing the line in clinical treatment of companion animals: recommendations from an ethics working party. Vet Rec. (2018) 182:664. 10.1136/vr.104559 29602799 PMC6035488

[36] Washington Administrative Code (WAC). Chapter 246-933 Veterinarians, Section 345 Client Communication Regarding Evaluation and Treatment. Olympia, WA: Washington Administrative Code (2020).

[37] AVMA. American Veterinary Medical Association Establishment and Use of Veterinary Clinical Studies Committees (VCSC). Available online at: https://www.avma.org/resources-tools/avma-policies/establishment-and-use-veterinary-clinical-studies-committees (accessed May 17, 2021).

[38] RCVS/BVA. Ethical Review for Practice-based Research: A report of a joint RCVS/BVA working party (2016). Available online at: https://www.rcvs.org.uk/document-library/rcvs–bva-ethical-review-working-party-report-2013. (accessed July 10, 2021).

[39] RCVS. Royal College of Veterinary Surgeons. Ethics Review Panel. Available online at: https://www.rcvs.org.uk/who-we-are/committees/standards-committee/ethics-review-panel/ (accessed July 10, 2021).

[40] FDA. Using a Centralized IRB Review Process in Multicenter Clinical Trials. Rockville, MD: U.S. Food and Drug Administration (2006).

[41] OHRP. Single IRB Exception Determinations. U.S. Health and Human Services. Rockville, MD: Office for Human Research Protections (2020).

[42] Moore SA McCleary-Wheeler A Coates JR Olby N London C. A CTSA One Health Alliance (COHA) survey of clinical trial infrastructure in North American veterinary institutions. BMC Vet Res. (2021) 17:90. 10.1186/s12917-021-02795-z 33632219 PMC7905595

[43] NIH. United States Public Health Service Policy on Humane Care Use of Laboratory Animals. Bethesda, MD: U.S. National Institutes of Health, Office of Laboratory Animal Welfare (NIH OLAW) (2015).

